# Clinical predictors of relapse and severe disease phenotype in children with non-systemic juvenile idiopathic arthritis

**DOI:** 10.1007/s00431-026-06842-5

**Published:** 2026-03-24

**Authors:** Doğacan Sarısoy, Fatma Aydın, Özen Taş, Onur Bahçeci, Betül Öksüz Aydın, Elif Erorhan, Tuğba Akkaya Hocagil, Zeynep Birsin Özçakar

**Affiliations:** 1 Department of Pediatrics, Division of Pediatric Rheumatology, Ankara University School of Medicine, Ankara, Turkey; 2 Department of Biostatistics, Ankara University School of Medicine, Ankara, Turkey

**Keywords:** Juvenile idiopathic arthritis, Prognosis, Relapse, Pediatric

## Abstract

The aim of this study was to identify the predictors of relapse and severe disease in non-systemic juvenile idiopathic arthritis (JIA), a heterogeneous childhood disease. Patients with JIA were grouped based on relapse status, and those with ≥ 2 relapses requiring biologics were classified as severe disease phenotype. A total of 142 patients (63.4% female) were included in the study. Seventy-three patients (51.4%) experienced at least one relapse after achieving remission, who were significantly characterized by female gender, younger age at diagnosis, positive ANA test, and longer disease duration. Ankle, elbow, metacarpophalangeal (MCP), and temporomandibular joint (TMJ) involvement was more prevalent in patients who had experienced at least one relapse. Longer disease duration, higher number of joints involved at the time of diagnosis, and MCP involvement were found as independent risk factors for relapse. Twenty patients (14%) were grouped as having a severe disease phenotype, characterized by a younger age at the time of diagnosis, longer disease duration, and a higher number of joints involved throughout the disease course. Younger age at diagnosis, ankle involvement, and TMJ involvement were found to be independent risk factors for a severe disease phenotype.

*Conclusion*: In our study, longer disease duration, MCP joint involvement, and a higher number of joints involved at the time of diagnosis were found to be associated with relapse in non-systemic JIA patients. On the other hand, younger age at diagnosis, ankle and TMJ involvement were associated with severe disease phenotype.

**Table:** 

What is Known: • JIA is a chronic joint disease characterized by relapses and remissions, and approximately half of patients experience at least one relapse after remission.	
What is New: • Multiple relapses and bDMARD requirement may define a severe disease course. Younger age, ankle, and TMJ involvement may predict severe disease course. • Relapses may occur with longer disease duration; not every relapse implies a severe disease course.

What is Known:

• JIA is a chronic joint disease characterized by relapses and remissions, and approximately half of patients experience at least one relapse after remission.

What is New:

• Multiple relapses and bDMARD requirement may define a severe disease course. Younger age, ankle, and TMJ involvement may predict severe disease course.

• Relapses may occur with longer disease duration; not every relapse implies a severe disease course.

## Introduction

Juvenile idiopathic arthritis (JIA) is defined as a chronic arthritis that manifests before the age of 16, persists for at least 6 weeks, and cannot be attributed to any other discernible cause. Traditionally, the disease is classified into seven subgroups: systemic arthritis, oligoarthritis, rheumatoid factor-negative polyarthritis, rheumatoid factor-positive polyarthritis, psoriatic arthritis, enthesitis-related arthritis, and undifferentiated arthritis [1]. However, new classifications have also emerged in recent years [2]. As the clinical courses of the groups are heterogeneous, predicting long-term outcomes and developing individualized treatment options is challenging.

Guidelines have been established to provide a framework for the pharmacological management of JIA. Although these guidelines have led to improvement in long-term outcomes, suboptimal results are still encountered during follow-up [3]. While some patients benefit from standard anti-rheumatic therapies and remain in remission, others require long-term and intensive treatment for recurring flare-ups.

Identifying risk factors associated with recurrence will lead to close follow-up of high-risk patients and inform the determination of treatments and their duration in these patients. Several risk factors have been identified such as a high Juvenile Arthritis Disease Activity Score (JADAS), increased erythrocyte sedimentation rate (ESR), involvement of the ankle, hip or cervical spine, the need for multiple intra-articular corticosteroid injections (IACI), and long disease duration. However, the majority of these studies have focused primarily on the oligoarthritis subgroup [4–6].

Defining numerous risk factors for each subgroup could complicate the definition of treatment and follow-up options. However, defining general risk groups could facilitate treatment plans for a significant proportion of patients and make it easier to develop personalized approaches for cases that do not fit into these groups.

The aim of this study is to identify clinical, laboratory, and demographic predictors of relapse in patients with non-systemic JIA, and to explore factors associated with a severe disease course requiring intense long-term treatment.

## Materials and methods

### Study design and participants

This retrospective observational study was conducted in patients diagnosed with JIA according to International League of Associations for Rheumatology (ILAR) criteria between June 2010 and June 2024 in our Pediatric Rheumatology Department. Patients were followed up and treated in accordance with JIA treatment guidelines [3]. Patients with a follow-up period of at least 6 months were included in the study. Those with missing data were excluded.

### Data collection

Data were retrieved from the patients’ electronic records. Demographic, clinical, and laboratory parameters such as age at diagnosis, gender, JIA subtype, affected joints, C-reactive protein (CRP), erythrocyte sedimentation rate (ESR), complete blood count parameters, antinuclear antibody (ANA), rheumatoid factor (RF), anti-citrullinated cyclic peptide (anti-CCP), HLA-B27, and treatment modalities such as non-steroidal anti-inflammatory drugs (NSAIDs), IACI, and conventional and biological disease-modifying anti-rheumatic drugs (cDMARDs and bDMARDs) were noted. JADAS27 scores were recorded at the time of diagnosis and 3, 6, 12, and 18 months of treatment.

### Definitions

The patients’ remission status was assessed according to the Wallace criteria. Remission criteria met for 6 consecutive months under treatment were considered “remission on medication” while remission criteria met during the first 12 months after treatment discontinuation were considered “remission off medication” [7]. Patients who experienced an exacerbation after meeting the remission criteria, on or off medication, were considered to have relapsed.

Patients were divided into two groups: those who experienced at least one relapse and those who did not. Demographic, clinical, and laboratory characteristics of the groups were compared. Patients were further categorized into distinct subgroups according to their relapse status and treatments they received. Patients who had ≥ 2 relapses and required biologic treatment were grouped as “severe (treatment dependent) disease phenotype” and compared with the rest of the patients.

#### Treatment and follow-up

Patients were followed up and treated in accordance with JIA treatment guidelines [3]. At diagnosis, all patients were started on NSAIDs, and suitable patients were given IACI. Patients who did not benefit from these first-line treatments, or for whom they had features of poor prognosis at diagnosis, were initiated on cDMARD treatments [3]. Patients who did not respond adequately to cDMARD therapy for at least 3 months were escalated to bDMARD therapy. According to our country’s health policies, at least 3 months of cDMARD therapy must be completed prior to bDMARD therapy. Therefore, all patients using bDMARDs had previously received cDMARD therapy for at least 3 months, and some of these patients had received combination therapy for a period (3–6 months). Following this, cDMARDs were often discontinued and treatment continued with a bDMARD.

#### Statistical analysis

Statistical analyses were performed using SPSS software version 25. Kolmogorov–Smirnov and Shapiro–Wilk tests were used to determine whether the variables were normally distributed. Descriptive analyses were presented as means and standard deviations for normally distributed variables, median and interquartile range for non-normally distributed variables, and frequencies for categorical variables. Student’s *t*-test was used for normally distributed variables, the Mann–Whitney *U* test was used for non-normally distributed and ordinal variables, and chi-square or Fisher’s exact tests were used for categorical variables. When the chi-square test indicated a significant association among categorical variables with more than two groups, post hoc pairwise comparisons were performed. Bonferroni correction was applied to adjust for multiple comparisons. For relapse analysis, significant independent variables (*p* < 0.05) in the univariate analysis were included in the multivariable logistic regression analysis. The odds ratios (OR) obtained in the adjusted regression analysis and their 95% confidence interval (CI) were calculated. A *p*-value less than 0.05 was considered a statistically significant result.

#### Ethics approval

This study was approved by the local ethics committee (Ankara University Ethics Committee of Human Research, Issue no: İ06–600–25), and it is performed in accordance with World Medical Association Declaration of Helsinki Ethical Principles for Medical Research Involving Human Subjects.

## Results

A total of 160 JIA patients were identified. Eighteen patients were excluded from the study: two due to severe comorbid diseases (one with acute lymphocytic leukemia and one with severe combined immunodeficiency), seven due to a diagnosis of systemic JIA, seven due to a follow-up period of less than 6 months, and two due to not meeting the criteria for remission during the follow-up period. Thus, a total of 142 patients were included in the study.

### Demographic, clinical, and laboratory features of patients at the time of diagnosis

Among 142 patients included in this study, 90 (63.4%) were female. Median (IQR) age at diagnosis was 6 (8) years. The median (IQR) follow-up period was 69 (65.25) months.

The majority of the patients were diagnosed with oligoarthritis (57.7%, *n* = 82). The most commonly affected joint was the knee (78.9%), followed by the ankle (33.1%). The median (IQR) ESR was 17.5 (31) mm/h, and median (IQR) CRP was 7.75 (25.8) mg/L at the time of diagnosis. The ANA test was positive in 79 patients (55.6%).

Demographic characteristics and clinical findings of patients with non-systemic JIA are given in Table 1.

**Table 1:** Demographic characteristics and clinical findings of patients

	*n* = 142
**Age at diagnosis,** years, median (IQR)	6 (8)
**Gender**, *n* (%)	
Female	90 (63.4)
Male	52 (36.6)
**Time between symptom onset and diagnosis,** months, median (IQR)	4 (9.25)
**Disease duration,** months, median (IQR)	69 (65.25)
**JIA subtypes**, *n* (%)	
Oligoarticular	82 (57.7)
Persistent	72 (50.7)
Extended	10 (7)
Polyarticular, rheumatoid factor negative	20 (14.1)
Polyarticular, rheumatoid factor positive	3 (2.1)
Enthesitis related arthritis	32 (22.5)
Psoriatic arthritis	3 (2.1)
Undifferentiated arthritis	2 (1.4)
**Number of affected joints**, median (IQR)	
At the time of diagnosis	2 (2)
First 6 months	2 (3)
Total	3 (4)
**Joint involvement,** *n* (%)	
Knee	112 (78.9)
Ankle	47 (33.1)
Wrist	32 (22.5)
Elbow	20 (14.1)
Metocarpophalangeal	16 (11.3)
Temporomandibular	12 (8.5)
**Uveitis,** *n* (%)	26 (18.3)
**JADAS 27 score,** median (IQR)	
Diagnosis	18 (5.65)
3 months	10 (12.8)
6 months	3 (10.25)
12 months	0 (0)
18 months	0 (0)

*JIA* Juvenile idiopathic arthritis, *JADAS* Juvenile Arthritis Disease Activity Score

### Comparison of patients who experienced at least one relapse and those who experienced no relapses

Sixty-nine patients (48.6%) did not have any relapses during the follow-up period while 73 patients (51.4%) experienced at least one relapse after achieving remission. Out of 73 patients, 51 (69.9%) experienced only one relapse while 22 (30.1%) experienced ≥ 2 relapses. Sixty-four (87.7%) patients experienced their first relapse after discontinuation of treatments, 28 (43.75%) of whom met the remission off medication criteria before relapse. Nine patients were still under medication at the time of relapse. Six patients were under cDMARD treatment, five achieved clinically inactive disease after IACI, while one needed a step-up in treatment. Three patients experienced relapses under bDMARD treatment, and all achieved clinically inactive disease with bDMARD switching. All of the bDMARD switchings were from a TNF inhibitor to another.

The female gender was significantly more prevalent among those who experienced at least one relapse (*p* = 0.012). The age at the time of diagnosis of patients who had experienced at least one relapse was found to be younger (*p* = 0.016), and their disease duration was found to be significantly longer (*p* < 0.001).

The number of joints involved at diagnosis, during the first 6 months, and throughout the duration of the disease was all significantly higher in patients with relapse (*p* = 0.011, 0.009, and < 0.001, respectively). ILAR subgroups were significantly different between groups (polyarticular and extended oligoarticular subgroups were predominant in the relapse group, *p* = 0.01). It was found that ankle, elbow, metacarpophalangeal (MCP), and temporomandibular joint (TMJ) involvement was more prevalent in patients who had experienced at least one relapse (*p* = 0.003, 0.001, 0.003, and 0.032, respectively). JADAS-27 score at the time of diagnosis was higher in patients who experienced at least one relapse (*p* = 0.014). Patients who experienced relapses had higher ESR and CRP levels at the time of diagnosis (*p* < 0.001 and *p* = 0.07, respectively). ANA positivity was significantly more prevalent among those who experienced at least one relapse (*p* = 0.02). The frequency of uveitis was higher in patients who experienced at least one articular relapse (*p* = 0.026).

Initial treatments used were significantly different between groups (*p* = 0.034). In post hoc pairwise analysis, cDMARD monotherapy was more frequently observed in the relapse group compared to cDMARD + bDMARD (Pearson *χ*^2^ = 4.56, *p* = 0.033); however, this difference did not remain statistically significant after Bonferroni correction for multiple comparisons (corrected *p* = 0.008). Treatment duration and time before achieving remission on or off medication were not significantly different between groups (*p* > 0.05).

Table 2 shows demographic, clinical, and laboratory comparisons between the group with at least one relapse and the group without.

**Table 2:** Comparison of patients with relapse and without relapse

	Relapse		*p*
	Yes *n* = 73	No *n* = 69	
**Gender**, *n* (%)			0.012*
Female	54 (74)	36 (52.2)	
Male	19 (26)	33 (47.8)	
**Age at diagnosis,** years, median(IQR)	5 (7)	8 (9)	0.016***
**Disease duration,** months, median (IQR)	83 (58)	48 (53)	< 0.001***
**JIA subtype**, *n* (%)			0.01**
Oligoartcicular, persistant	32 (43.8)	40 (58)	
Oligoarticular, extended	9 (12.3)	1 (1.4)	
Poliarticular, RF +	3 (4.1)	0 (0)	
Poliarticular, RF –	12 (16.14)	8 (11.6)	
Enthesitis related	13 (17.8)	19 (27.5)	
Psoriatic	3 (4.1)	0 (0)	
Undifferantiated	1 (1.4)	1 (1.4)	
**Joint involvement**, *n* (%)			
Knee	62 (84.9)	50 (72.5)	0.107*
Ankle	33 (45.2)	14 (20.3)	0.003*
Elbow	17 (23.3)	3 (4.3)	0.001**
Wrist	20 (27.4)	12 (17.4)	0.22*
MCP	14 (19.2)	2 (2.9)	0.003**
TMJ	10 (13.7)	2 (2.9)	0.032**
**Number of joints involved**, median (IQR)			
At diagnosis	2 (3)	1 (1)	0.011***
First 6 months	2 (4)	1(1)	0.009***
Total	4 (5)	2 (2)	< 0.001***
**JADAS-27 score**, median (IQR)			
Diagnosis	19 (7.9)	17 (5.1)	0.014***
3 months	11 (10.3)	9 (13)	0.22***
6 months	3 (9.6)	0 (11)	0.54***
12 months	0 (0.6)	0 (0)	0.57***
18 months	0 (0)	0 (0)	0.12***
**Uveitis,** *n* (%)	19 (26)	7 (10.1)	0.026*
**ESR**, mm/h, median (IQR)	29 (42)	12 (16)	< 0.001***
**CRP**, mg/L, median (IQR)	10.1 (24.2)	5.4 (25.5)	0.07***
**ANA positivity**, *n* (%)	48 (65.8)	31 (44.9)	0.02*
**RF positivity**, *n* (%)	3 (4.3)	0 (0)	0.245**
**Anti-CCP positivity**, *n* (%)	4 (16.7)	1 (7.7)	0.638**
**HLA B27 positivity**, *n* (%)	12 (21.4)	18 (35.3)	0.168*
**Initial treatments used**, *n* (%)			
NSAID/IACI	14 (19.2)	18 (26.1)	0.034*
cDMARD only	43 (62.3)	26 (37.7)	
bDMARD only	0 (0)	2 (2.9)	
cDMARD + bDMARD	16 (21.9)	23 (33.3)	
**Treatment duration,** months, median (IQR)			
cDMARD only	20 (20)	17 (7.5)	0.17***
bDMARD only	-	26	-
cDMARD + bDMARD	25 (18)	28.5 (16.25)	0.88***
**Time to remission on medication**, months, median (IQR)	9 (6)	9 (6)	0.37***
**Remission off medication rates**, *n* (%)	28 (38.4)	38 (55.1)	0.046*
**Time to remission off medication**, months, median (IQR)	27.5 (18)	25 (20)	0.59***

*JIA* juvenile idiopathic arthritis, *MCP* metacarpophalangeal joint, *TMJ* temporomandibular joint, *JADAS* Juvenile Arthritis Disease Activity Score, *ESR* erythrocyte sedimentation rate, *CRP* C-reactive protein, *ANA* anti-nuclear antigen, *RF* rheumatoid factor, *anti-CCP* anti-cyclic citrullinated peptide, *HLA* human leucocyte antigen, *NSAID* non-steroidal anti-inflammatory drugs, *IACI* intraarticular corticosteroid injection, *DMARD* disease modifying anti-rheumatic drugs, *bDMARD* biologic-DMARDs

^*^Pearson chi-square, **Fisher’s exact test, ***Mann–Whitney *U* test

A multivariable logistic regression model was used to examine predictors of the relapse. Longer disease duration was significantly associated with increased relapse rate (OR = 1.021, 95% CI [1.007, 1.038], *p* = 0.007). MCP involvement was also associated with increased relapse rate (OR = 12.4, 95% CI [1.822, 172.9], *p* = 0.024). Additionally, higher number of joints involved at the time of diagnosis predicted higher risk of relapse (OR = 1.69, 95% CI [1.11, 2.72], *p* = 0.021). In order to enhance the reliability of the analysis, JIA subgroups were recategorised into three groups: oligoarthritis, enthesitis-related arthritis (ERA), and others. “Other” patient group was selected as the reference group in the regression analysis. Patients in the oligoarthritis group had substantially lower risk of relapse compared to the others group (OR = 0.04, 95% CI [0.001, 0.679], *p* = 0.043) (Table 3).

**Table 3:** Factors associated with relapse in JIA

	OR	95% CI	*p*
**Gender,** male	0.76	[0.242, 2.358]	0.63
**Age at diagnosis**	1.03	[0.901, 1.182]	0.664
**Disease duration**	1.02	[1.007, 1.038]	**0.007**
JIA group: oligoarthritis	0.04	[0.001, 0.679]	**0.043**
JIA group: ERA	0.77	[0.168, 3.204]	0.725
**Joint involvement**			
Ankle involvement	1.46	[0.404, 5.216]	0.556
Elbow involvement	7.23	[0.751, 117.499]	0.12
MCP involvement	12.4	[1.822, 172.190]	**0.024**
TMJ involvement	2.6	[0.362, 26.589]	0.363
**Uveitis**	3.63	[1.000, 13.826]	0.051
**ANA**, positive	1.471	[0.516, 4.229]	0.468
**Higher number of joints,** at the time of diagnosis	1.69	[1.116, 2.728]	**0.021**
**JADAS-27** at diagnosis	0.96	[0.821, 1.123]	0.602
**CRP**	0.99	[0.966, 1.015]	0.558

*JIA* juvenile idiopathic arthritis, *JADAS* Juvenile Arthritis Disease Activity Score, *CRP* C-reactive protein, *ANA* anti-nuclear antigen, *ERA* enthesitis-related arthritis, *MCP m*etacarpopalangeal joint, *TMJ* temporomandibular joint

### Comparison of patients with “severe (treatment dependent) disease phenotype” and others

**Fig. 1:**
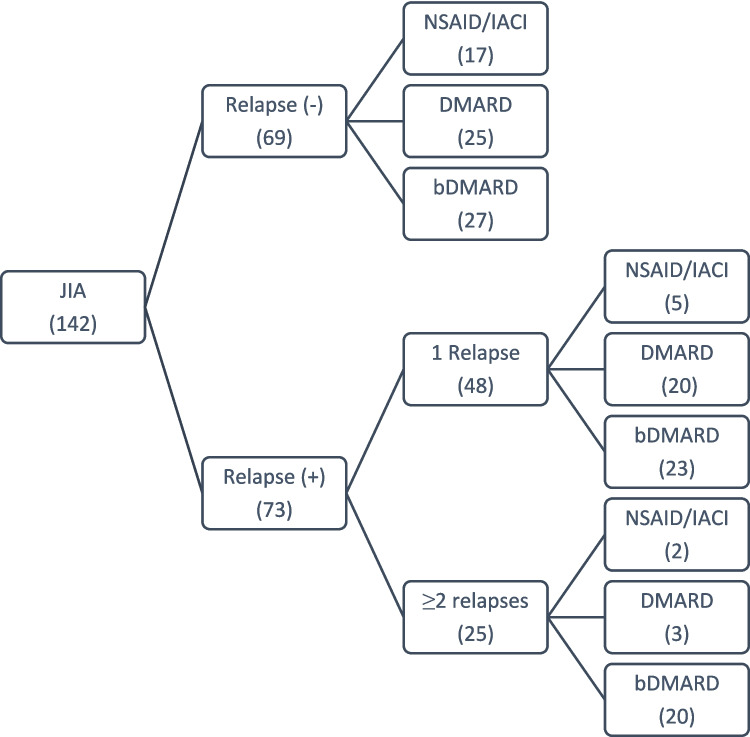
Relapse status of patients and treatments used. NSAID, non-steroidal anti-inflammatory drugs; IACI, intra-articular steroid injection; cDMARD, conventional disease-modifying anti-rheumatic drugs; bDMARDs, biologic disease-modifying anti-rheumatic drugs

Patients were further categorized into distinct subgroups according to their relapse status and treatments they received. Relapse status of patients and treatments used in each group are shown in Fig. 1.

Twenty patients (13.8%) had ≥ 2 relapses and required bDMARD treatment, which were defined as “severe disease (treatment dependent) phenotype.” These patients were compared with the rest of the patients.

The age at the time of diagnosis of the “severe (treatment dependent) disease phenotype” group was found to be significantly younger (*p* = 0.008), and their disease duration was found to be significantly longer (*p* < 0.001). The number of joints involved throughout the duration of the disease was significantly higher in the “severe disease phenotype” group (*p* = 0.001). ILAR subgroups were not different between groups. It was found that ankle and TMJ involvement were more prevalent (*p* < 0.001 and 0.015, respectively), and the JADAS-27 score at the time of diagnosis, ESR and CRP were higher (*p* = 0.005, < 0.001 and 0.022, respectively) in the “severe (treatment dependent) disease phenotype” group (Table 4).

**Table 4:** A comparison of patients with severe (treatment dependent) disease phenotype and others

	Severe disease phenotype *n* = 20	Others *n* = 122	*p*
**Gender**, *n* (%)			
Female	15 (75)	75 (61.5)	0.36*
Male	5 (25)	47(38.5)	
**Age at diagnosis,** years, median(IQR)	4 (5)	6.5 (8)	0.008***
**Disease duration,** months, median (IQR)	99.5 (53.5)	60 (57.5)	< 0.001***
**JIA subtype**, *n* (%)			
Oligoartcicular, persistant	7 (35)	65 (53.3)	0.11**
Oligoarticular, extended	4 (20)	6 (4.9)	
Poliarticular, RF +	0 (0)	3 (2.5)	
Poliarticular, RF –	4 (20)	16 (13.1)	
Enthesitis related	4 (20)	28 (23)	
Psoriatic	0 (0)	3 (2.5)	
Undifferentiated	1 (5)	1 (0.8)	
**Joint involvement**, *n* (%)			
Knee	17 (85)	95 (77.9)	0.56**
Ankle	14 (70)	33 (27)	< 0.001*
Elbow	6 (30)	14 (11.5)	0.063*
Wrist	5 (25)	27 (22.1)	1*
MCP	3 (15)	13 (10.7)	0.7**
TMJ	5 (25)	7 (5.7)	0.015*
**Number of joints involved**, median (IQR)			
At diagnosis	2 (3.2)	2 (2)	0.47***
First 6 months	2.5 (4.2)	2 (2)	0.6***
Total	5 (4)	2 (3)	0.001***
**JADAS-27 score**, median (IQR)			
Diagnosis	21.3 (8.4)	17 (5.1)	0.005***
3 months	12 (9.6)	10 (13.4)	0.476***
6 months	5.4 (10.9)	2.5 (10)	0.465***
12 months	0 (3.2)	0 (0)	0.173***
18 months	0 (0)	0 (0)	0.882***
**Uveitis**, *n* (%)	7 (35)	19 (15.6)	0.077*
**ESR**, mm/sa, median (IQR)	52 (34.5)	16 (23)	< 0.001***
**CRP**, mg/L, median (IQR)	18 (29.4)	6.2 (22.9)	0.022***
**ANA positivity**, *n* (%)	15 (75)	64 (52.5)	0.101*
**RF positivity**, *n* (%)	0 (0)	3 (2.6)	1**
**Anti-CCP positivity**, *n* (%)	1 (12.5)	4 (13.8)	1**
**HLA B27 positivity**, *n* (%)	5 (27.8)	25 (28.1)	1**

*JIA *juvenile idiopathic arthritis, *MCP* metacarpophalangeal joint, *TMJ* temporomandibular joint, *JADAS* Juvenile Arthritis Disease Activity Score, *ESR* erythrocyte sedimentation rate, *CRP* C-reactive protein, *ANA* anti-nuclear antigen, *RF* rheumatoid factor, *anti-CCP* anti-cyclic citrullinated peptide, *HLA* human leukocyte antigen

^*^Pearson chi-square, **Fisher’s exact test, ***Mann–Whitney *U* test

A binary logistic regression was conducted to examine predictors of being in the “severe (treatment dependent) disease phenotype” group compared to all other groups. Lower age at diagnosis was significantly associated with higher risk of being in the severe disease phenotype group (OR = 1.28, 95% CI [1.03–1.67], *p* = 0.046). Patients with ankle and TMJ involvement had significantly higher risk of being in the “severe disease phenotype” group compared to those without involvement (OR = 6.12, 95% CI [1.60, 27.78], *p* = 0.012 and OR = 8.18, 95% CI [1.17, 68.82], *p* = 0.039, respectively) (Table 5).

**Table 5:** Factors associated with severe (treatment dependent) disease phenotype in JIA patients

	OR	95% CI	*p*
**Age at diagnosis**	1.28	[1.03, 1.67]	**0.046**
**Disease duration**	1.02	[1.00, 1.04]	0.072
**Joint involvement**			
Ankle involvement	6.12	[1.60, 27.88]	**0.012**
TMJ involvement	8.18	[1.17, 68.82]	**0.039**
**Number of joints,** total	1.02	[0.84, 1.22]	0.81
**JADAS-27** at diagnosis	1.05	[0.90, 1.23]	0.54
**ESR**	1.02	[0.99, 1.05]	0.27

*TMJ* temporomandibular joint, *ESR* erythrocyte sedimentation rate, *JIA* juvenile idiopathic arthritis

## Discussion

Children with JIA can sometimes be difficult to diagnose, but the disease is relatively easy to control once treatment has begun, particularly given recent advances in the biological area. However, the most challenging issues are how long treatments should last, when to withdraw them, and how to manage flare-ups in this chronic, recurrent disease. It would be helpful for clinicians to have some clinical indicators to predict the course of the disease. In our cohort, more than half of the patients experienced at least one relapse after achieving remission. These relapses were associated with female gender, younger age at diagnosis, longer disease duration, higher joint involvement counts, and certain JIA subtypes. The relapse group was further characterized by higher baseline JADAS-27, ESR, ANA positivity, uveitis, and specific joint involvement. In addition, the severe (treatment dependent) disease phenotype was found to have younger age at diagnosis, longer disease duration, more joint involvement overall, and more frequent ankle and TMJ involvement. Together, these findings emphasize the significance of early disease characteristics and specific joint patterns in predicting the risk of relapse and the progression of severe disease.

The rates of flare-up after remission vary in patients with JIA. Many studies have shown that the relapse rate ranges from 26.3% to 100% [8–14]. This discrepancy may be due to a variety of factors, including small sample sizes, heterogeneous patient groups, and different definitions of relapse. In our study, approximately half of the patients (51.4%) experienced at least one relapse after achieving remission on or off medication. The relapse rate in the ReAChOut cohort, which included all JIA subgroups, was comparable to that observed in our study, with relapses occurring in 54.6% of patients [15]. These results suggest that, during follow-up, it should be taken into account that half of the patients may experience at least one exacerbation after achieving remission.

The present study found that female gender and younger age at diagnosis were associated with relapse, but only younger age at diagnosis was associated with a severe (treatment dependent) disease phenotype. Tuomi et al. also suggested that male gender and the absence of uveitis in the oligoarthritis group predicted remission [16]. Contrary to our study, Aquilani et al. demonstrated a higher relapse rate in male patients. However, no significant relationship was found between gender and relapse frequency in many other studies [8, 12, 13]. Lovell et al. demonstrated a negative correlation between age at diagnosis and relapse rates, which is consistent with our findings. However, this correlation was not observed in different cohorts [8, 9, 13, 14].

In our study, higher JADAS-27 scores, a higher number of joints involved, higher ESR at diagnosis, and positive ANA were associated with relapse. Higher baseline JADAS-27 scores were also associated with a severe disease phenotype. Van Dijkhuisen et al. demonstrated that patients with oligoarthritis who had higher JADAS 71 scores at diagnosis were less likely to achieve clinically inactive disease [17]. Similarly, Sezer et al. reported that lower JADAS-27 scores were associated with a higher chance of achieving clinically inactive disease within 3 months, and that patients who experienced relapses had a higher ESR, which is consistent with our study [5]. In contrast to our study, Aquilani et al. showed that there was no correlation between the number of joints involved at baseline and relapse status. However, they found a significant difference in relapse rates between ANA-positive and ANA-negative groups, which is compatible with our results [8]. As the presence of ANA has been suggested to indicate an immunologically active disease, it is thought that it may also be related to flare-ups [18]. However, Simonini et al. found that ANA was not significant in predicting relapse [19].

JIA is a highly heterogeneous disease consisting of seven subgroups according to the ILAR classification. The clinical findings and disease course of each subgroup vary, and therefore, the frequency of disease exacerbations can be expected to differ between groups. However, in some previous studies, no significant relationship was found between ILAR subgroups and relapse [12, 13]. In our cohort, polyarticular or extended oligoarticular JIA subtypes were significantly more common in the relapse group. Furthermore, multivariate regression analysis revealed that the oligoarthritis subgroup (*both persistent and extended*) had lower odds of experiencing relapses. The absence of precise results on this issue could encourage discussions about the need for a new JIA classification that encompasses more homogeneous groups than the ILAR classification [2].

In our cohort, ankle, elbow, MCP, and TMJ involvements were more prominent in the relapse group, and multivariate regression analysis revealed that MCP involvement was an independent risk factor of relapse. On the other hand, ankle and TMJ involvement were significantly more common in the severe disease phenotype group, and both were shown to be independent risk factors for severe disease phenotype. Similar to our cohort, Sezer et al.’s study also revealed a higher relapse risk for patients with ankle involvement [5]. Esbjörnsson et al. demonstrated that patients with ankle involvement were less likely to be clinically inactive at the 8-year follow-up duration [20]. TMJ involvement is often asymptomatic and may consequently be underdiagnosed. Artamonov et al. demonstrated that TMJ involvement was associated with longer disease duration, longer time to achieve remission, and the need for more frequent bDMARD therapy [21]. These results suggest that which joint is affected is related to the prognosis. Patients with ankle and TMJ involvement should be closely monitored. During follow-up, it should be kept in mind that TMJ involvement may be clinically silent. If there is any suspicion, the appropriate imaging methods should be performed.

In our study, we found a significant correlation between disease duration and relapse frequency. Other studies have also reported a positive correlation between relapse rates and follow-up duration [4, 8]. While the risk of relapse increases with disease duration, there is substantial evidence to suggest that restarting treatment in cases of relapse results in a favorable clinical response [10, 12].

This study has several limitations, including being a single-center and retrospective analysis. However, considering the inconsistencies in the literature, we believe that our results will be very useful during patient follow-up.

In conclusion, approximately half of patients diagnosed with JIA experience at least one relapse after achieving remission. A higher number of joints affected at the time of diagnosis, a longer duration of the disease, and MCP joint involvement are useful predictors of exacerbation of the disease; therefore, patients exhibiting these features should be closely monitored for relapse. While the overall relapse rate is high among JIA patients, a severe disease phenotype characterized by multiple relapses and the need for bDMARD therapy was observed in only around 14% of cases. This severe phenotype appears to be associated with a younger age at diagnosis and with ankle and TMJ involvement. Therefore, while many patients experience disease flares over time, not every relapse indicates a severe clinical course.

The focus of future research should be an objectively measurable biomarker or a radiological scoring system to predict prognosis. However, despite ongoing research in this area, there is still a need for indicators applicable to daily practice.

## Data Availability

No datasets were generated or analysed during the current study.
